# Identification of the Constituents of Ethyl Acetate Fraction from *Smilax china* L. and Determination of Xanthine Oxidase Inhibitory Properties

**DOI:** 10.3390/ijms24065158

**Published:** 2023-03-08

**Authors:** Xin Li, Shanshan Liu, Weili Jin, Wenkai Zhang, Guodong Zheng

**Affiliations:** Jiangxi Key Laboratory of Natural Product and Functional Food, College of Food Science and Engineering, Jiangxi Agricultural University, Nanchang 330045, China

**Keywords:** *Smilax china* L. extract, xanthine oxidase, ethyl acetate fraction, competitive inhibitor, HPLC–MS

## Abstract

The aim of this work was to investigate the xanthine oxidase (XO)-inhibitory activity of ethanol extracts from *Smilax china* L. and to identify the active compounds in the ethyl acetate (EtOAc) fraction. Extraction of ethanol extracts from *Smilax china* L. and then ethanol extracts were concentrated, and the polyphenolic compounds were extracted with petroleum ether (PE), chloroform, EtOAc, n-butanol (n-BuOH), and residual ethanol fractions. Their effects on XO activity were then compared separately. The polyphenolic components of the EtOAc fraction were identified by HPLC and HPLC–mass spectrometry (HPLC-MS) analysis. Kinetic analysis demonstrated that all these extracts showed XO-inhibitory properties, and among them the EtOAc fraction had the strongest inhibitory effect (IC_50_ = 101.04 μg/mL). The inhibitory constant (*K*_i_) of the EtOAc fraction on XO activity was 65.20 μg/mL, showing excellent inhibition on XO in the competitive mode. Sixteen compounds were identified from the EtOAc fraction. The study demonstrates that the EtOAc fraction of *Smilax china* L. may be a potential functional food to inhibit XO activity.

## 1. Introduction

The pathological feature of gout is the precipitation of monosodium urate crystals in tendons, kidneys, joints, and surrounding tissues, resulting in painful acute gouty arthritis [1]. Hyperuricemia is usually related to the overproduction of uric acid, leading to an enhanced serum level of uric acid, which is due to elevated xanthine oxidase (XO) activity. XO is a molybdenum (Mo)-containing enzyme that catalyzes the oxidation of purine bases to uric acid and produces reactive oxygen and nitrogen species [2,3,4]. Excessive amounts of reactive oxygen species in the body can increase oxidative stress, causing a variety of pathological processes, such as inflammation, atherosclerosis, and cancer [5,6]. Therefore, XO inhibitors are regarded as the main drugs for treating these diseases. Although the therapeutic agent (i.e., allopurinol) is the primary inhibitor in the treatment of gout, it exhibits side effects including hepatotoxicity, Stevens Jones syndrome, and nephropathy [7,8,9]. It has been demonstrated that numerous food and medicinal herbs that suppress XO can be used to prevent or treat gout [10]. Furthermore, phenolics have been shown to be strong XO inhibitors [11]. The therapeutic effects of numerous plants have been ascribed to polyphenol because of its antioxidant and enzyme-inhibitory properties [12,13]. For example, apigenin is a strong XO inhibitor, with an inhibition constant (*K*_i_) value of 0.61 ± 0.31 μM [14]. Myricetin was reported to inhibit uric acid formation in a mixed-type manner with an IC_50_ of (8.66 ± 0.03) ×10 ^−6^ mol/L [15]. Dong et al. found that pinobanksin and galangin inhibited XO activity with IC_50_ values of 1.37 × 10^−4^ and 1.63 × 10^−4^ mol/L, respectively [16]. Moreover, kaempferol has been found to reversibly inhibit the activity of XO in a competitive manner with a *K*_i_ of 6.77 ± 1.02 μM [17]. 

*Smilax china* L., also called “Jingangteng (JGT)” and “Baqia”, is primarily derived from Liliaceae plants. It is cultivated throughout East China, Central and South China, and Southwest China. This plant is listed in the Chinese pharmacopoeia and generally used to treat pelvic inflammatory disease, diuresis, rheumatoid arthritis, detoxication, tumors, and other diseases; it is commonly used in traditional Chinese medicine, functional soup, and dessert food in China [18,19]. Extensive previous studies on *Smilax china* L. have revealed that it has anti-inflammatory [20], anti-obesity [21,22], anti-bacterial [23], and other activities. Phytochemical investigations have shown that steroid saponins and polyphenols are the major chemical constituents of *Smilax china* L. which show beneficial pharmacological effects [24,25]. This study aimed to explore the XO-inhibitory effect of ethyl acetate (EtOAc) of *Smilax china* L. and its chemical composition. 

In this study, the ethanol extract was partitioned successively by petroleum ether (PE), chloroform, EtOAc, and n-butanol (n-BuOH) according to solvent polarity. These extracts’ XO-inhibitory effects were investigated. Among them, the EtOAc extract exhibited the strongest XO-inhibitory activity, and its components were further identified by HPLC-MS.

## 2. Results and Discussion

### 2.1. The Contents of Total Phenolic and Total Flavonoid

**Figure 1:**
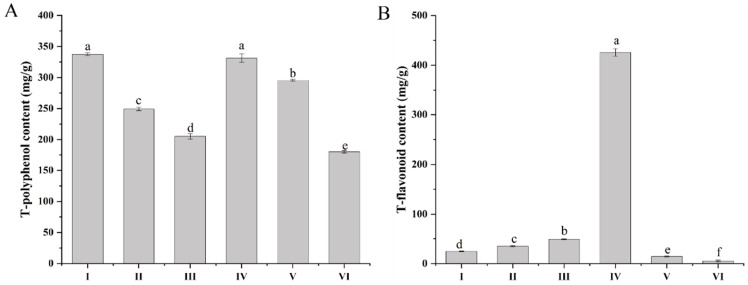
The contents of flavonoids and polyphenols in different extraction fractions. I, ethanol extract; II, PE fraction; III, chloroform fraction; IV, EtOAc fraction; V, n-BuOH fraction; VI, residual ethanol solution fraction. (**A**) Total polyphenol content; (**B**) Total flavonoid content; Different letters (a–f) represent significant differences among groups (*p* < 0.05). Values are expressed as the mean ± SD.

All extracts/fractions of Smilax china L. were measured for their total contents of phenolics and flavonoids, as shown in Figure 1. Among these extracts, the ethanol extract (337.612 ± 2.13 mg/g) contained highest number of phenolic compounds, followed by the EtOAc (331.47 ± 6.835 mg/g), n-BuOH (295.832 ± 1.11 mg/g), PE (249.301 ± 2.738 mg/g), chloroform (205.282 ± 4.356 mg/g), and residual ethanol (180.113 ± 1.846) fractions. In addition, the total flavonoid content was found to be highest in the EtOAc fraction (425.90 ± 7.315 mg/g). These data suggested that different solvent partitions successfully distinguished different-polarity polyphenols and flavonoids.

### 2.2. Effect of Smilax china L. Extracts on XO Activity

**Figure 2:**
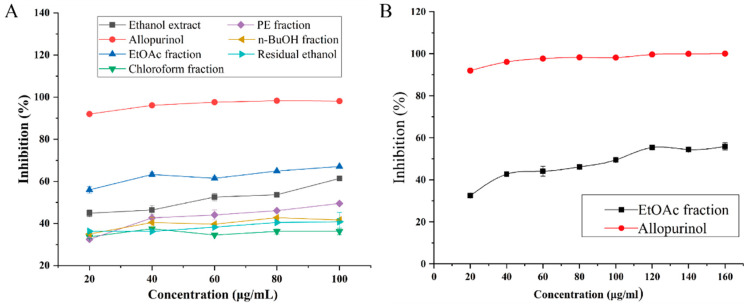
(**A**) XO-inhibitory activity of *Smilax china* L. extracts. (**B**) Inhibitory effects of the EtOAc fraction and allopurinol on XO activity.

As presented in Figure 2A, the XO-inhibitory effects of the ethanol extract (EtOH) and its extracted fractions, including PE, chloroform, EtOAc, n-BuOH, and residual ethanol solution, were compared. The XO-inhibitory effect of the extracts was reduced in the following order: EtOAc fraction > ethanol extract > PE fraction > n-BuOH fraction > residual ethanol > chloroform fraction, indicating that the extracts were potential XO inhibitors. Allopurinol is used as a XO-inhibitory drug, and its inhibitory activity against XO is stable around 90% at a concentration of 20–100 μg/mL. The EtOAc fraction showed the best XO-inhibitory effect, and further linear fitting was carried out. The equation between inhibition and concentration is Y = 54.00565 − 3.52195X + 0.18884X^2^ − 0.00411X^3^ + (4.3291 × 10^−5^)X^4^ – (2.18682 × 10^−^7)X^5^ + (4.23862 × 10^−10^)X^6^ (R^2^ = 0.9914), where Y = 50 and X = 101.04004. Thus, the IC_50_ value of the EtOAc fraction is 101.04004 μg/mL. Among them, the EtOAc extract had the best inhibitory activity on XO, demonstrating that the XO-inhibitory activity was efficiently enriched in the EtOAc fraction Additionally, the inhibitory effects of the EtOAc fraction and allopurinol on XO were compared (Figure 2B). The data indicated that a new, potent inhibitor could be explored in the EtOAc fraction.

### 2.3. The Type of Inhibition by Lineweaver–Burk Plot Analysis

**Figure 3:**
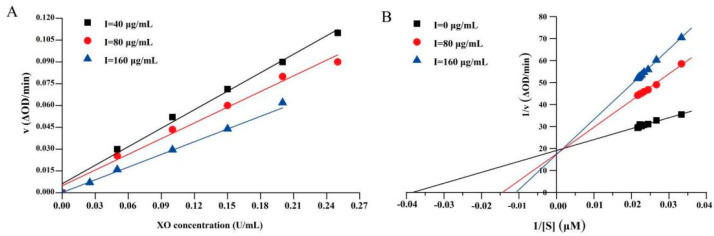
Inhibition kinetics of XO by EtOAc fraction. (**A**) Plots of v vs. [XO]. c (XO) = 150 μM, c (EtOAc fraction) = 40, 80, and 180 μg/mL. (**B**) Lineweaver–Burk plots. c (XO) = 0.1 U/mL, c (EtOAc fraction) = 0, 80, and 160 μg/mL.

To measure whether the inhibitory activity of the EtOAc fraction on XO is reversible, plots of ν versus [XO] at various concentrations were constructed in Figure 3A. As the concentration of the EtOAc fraction increased, all the lines passed through the origin, and the slopes decreased. The results indicated that the inhibitory effect of the EtOAc fraction on XO was reversible and that there was non-covalent intermolecular interaction with XO. Furthermore, the kinetic mechanism of the EtOAc fraction was investigated using a double-reciprocal Lineweaver–Burk plot according to Equation (1).

As illustrated in Figure 3B and Table 1, the horizontal axis intercept (−1/*K_m_*) gradually increased with increasing EtOAc fraction concentration, while the vertical axis intercept (1/*V_max_*) remained constant, indicating that the EtOAc fraction was a competitive XO inhibitor. This was possible because the EtOAc fraction was connected directly to the active position of XO, blocking entry to the enzyme active site and resulting in an obvious reduction in the XO-inhibitory activity. Furthermore, the secretion of uric acid is decreased, but not sufficiently to render XO inactive. Thus, dialysis, ultrafiltration, and other methods are still used to restore enzyme activity [26]. According to Equation (2), the *K_i_* value was 65.20 μg/mL, and the concentration reached 160 μg/mL. The lower the *K_i_* value, the tighter the binding to XO, and consequently the greater the inhibitory effect on XO [27].

### 2.4. Identification of Active Compounds

**Figure 4:**
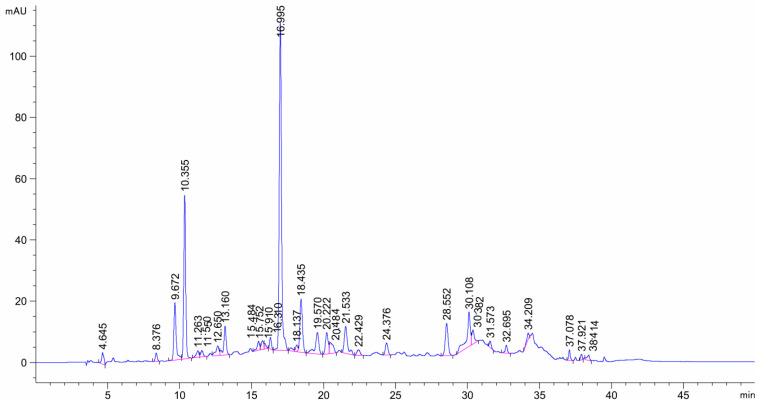
HPLC chromatograms of the EtOAc fraction.

**Figure 5:**
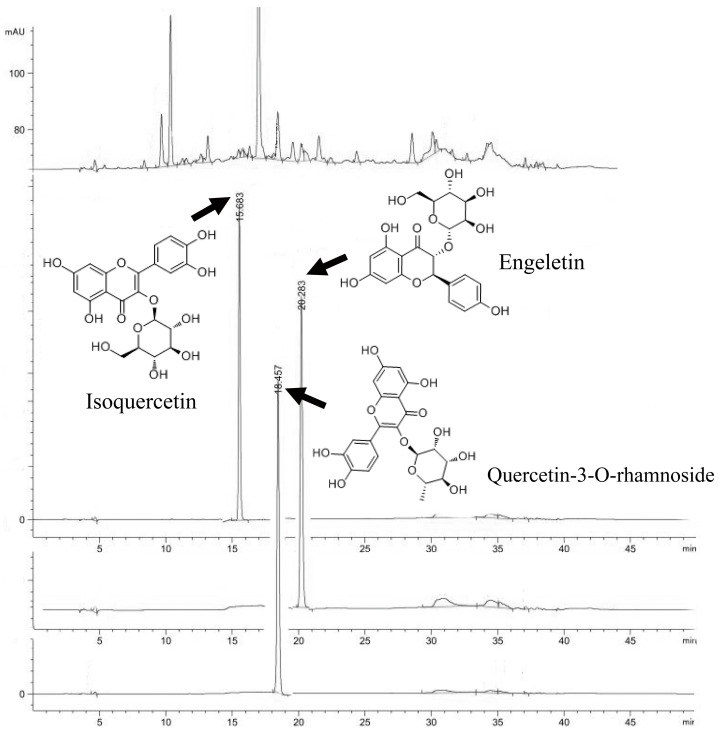
HPLC chromatograms by comparison with the standards.

Smilax china L. extracts were effective against XO, and the EtOAc fraction was the most effective, which might be related to the active components, such as polyphenols. Thus, it is very necessary to identify the components of the EtOAc fraction. Therefore, the composition of the EtOAc fraction was determined by applying the HPLC chromatography method (Figure 4). Quercetin, quercetin-3-O-rhamnoside, engeletin, rutin, chlorogenic acid, gallic acid, kaempferol, kaempferitrin, isoquercetin, and astragalin were used as standards. As shown in Figure 5 and Table 2, the polyphenols were identified based on the retention time and the chromatographic peaks of standards. The main components were identified as isoquercetin (0.950%), quercetin-3-O-rhamnoside (6.574%), and engeletin (2.461%). In vivo studies have also evidenced that polyphenol compounds exhibit a significant effect on XO activity [13]. Huang et al. [28] discovered that puerarin, myricetin, morin, apigenin, kaempferol, and quercetin could significantly lower the serum uric acid level in hyperuricemic rats, and some of them also inhibited liver XO activity. Therefore, the results highlighted the potential of polyphenols to treat gout, illustrating that the EtOAc fraction, which was rich in XO inhibitors, is an active ingredient that deserves further investigation.

### 2.5. Chemical Constituent Analysis by HPLC-MS

**Figure 6:**
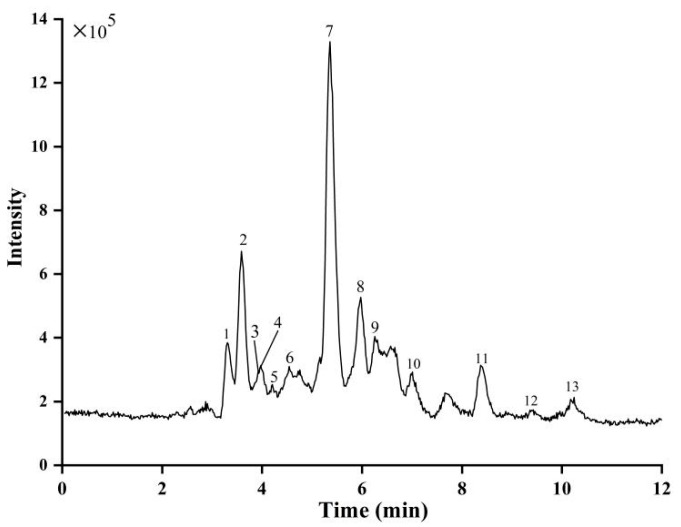
The total ion chromatogram (TIC) of the EtOAc fraction by HPLC-MS.

Compounds were tentatively identified according to precursor ions, fragment ions, the corresponding reference compound, and the literature and databases at http://www.massbank.jp/ (accessed on 8 May 2019), http://www.chemspider.com/ (accessed on 11 May 2019), and https://scifinder.cas.org/ (accessed on 13 May 2019). The retention times (Rt), observed and calculated mass er.

rors (ppm), molecular formulas, MS/MS product ions, and proposed compounds are summarized in Table 3. The total ion chromatogram (TIC) is depicted in Figure 6. Thirteen compounds were identified and tentatively characterized.

By comparing the retention time, MS, and MS/MS fragmentation pattern with the related literature, peak **1** was detected; the precursor ion [M−H]^−^ was located at *m*/*z* 353.085 (C_16_H_18_O_9_), the major fragment ion was located at *m*/*z* 191.055, and referring to the literature, it was identified as chlorogenic acid (quinic acid) [27]. Research has shown that chlorogenic acid protects against tissue damage caused by ischemia/reperfusion, enhances antioxidant activity, and scavenges superoxide free radicals. In addition, chlorogenic acid interacts with enzymes, altering their structure and biological activity [29]. The production of reactive oxygen species (ROS) occurs during the process of XO catalyzing purine to generate uric acid. When XO activity is elevated, it will result in a large amount of ROS generation, causing oxidative damage in the body, which is related to the onset of ischemia–reperfusion injury [23]. Reactive oxygen species (ROS) are the main cause of organ damage after the blood supply to the ischemic tissue is restored. Therefore, the results indicated that the inhibitory effect of chlorogenic acid on XO needs further study.

Peaks **2** and **5** revealed [M−H]^−^ ions located at *m/z* 329.086 and 329.086, respectively. The MS^2^ spectrum showed that the characteristic vanillic acid molecular ions were at *m/z* 167.035 and 167.033. According to literature data from [27], peaks **2** and **5** were vanillic acid hexoside. Peak 3 was (−)-epicatechin or catechin-deprotonated pseudo-molecular ion; MS was *m*/*z* 289, and MS^2^ was *m/z* 121.026 [30,31].

Peaks **4** and **6** with an identical molecular formula (C_46_H_28_O_10_) were tentatively proposed as catechin-O-digalloyl-C-rhamnoside and its isomers based on the presence of a fragment ion at *m/z* 740.168, resulting in an [M−H]^−^ ion at *m/z* 739.161. Thus, the peaks were characterized as catechin-O-digalloyl-C-rhamnoside (peak **4**), and peak **6** should be identified as an isomer of catechin-O-digalloyl-C-rhamnoside by comparison with the literature [31]. 

Peaks **7** and **8** in the spectrum had the same molecular ion [M−H]^−^, which had an *m/z* value of 353. A major fragment at 450.116 and 450.110, respectively, was also observed for both peaks. Thus, the peaks were characterized as eriodictyol-C-hexose (peak **7**) and eriodictyol-C-hexose isomers (peak **8**), which are isomers. According to the literature, it was tentatively identified as eriodictyol-C-hexose [32].

Peaks **9** and **11** gave the same precursor ion at [M−H]^−^ *m/z* 451.099 and MS^2^ fragments at *m/z* 341.063 with the molecular formula C_24_H_20_O_9_. These structural isomers exhibited different retention times to enable the identification of cinchonain I (peak **9,** Rt = 6.26 min) and cinchonain I isomers (peak **11**, Rt = 8.37 min) [33]. Another compound (peak **12**) had a molecular ion [M−H]^−^ at *m/z* 613.128 and was therefore tentatively identified as cinchonain I hexose, but it presented a different fragment from peaks **9** and **11**.

Peak **10** with [M−H]^−^ in the negative mode at *m/z* 243.064 and an MS^2^ fragment ion at 175.073 was identified as uridine.

Peak 13 with [M−H]^−^ at *m/z* 227.068 and an MS^2^ ion at 143.048 was tentatively identified as (2)-2-(aspartylamino)-4-pentynoic acid by comparison with the reference spectral fragments.

## 3. Discussion

Gout, also referred to “the disease of kings” or “rich man’s disease”, is a classical disease that is now recognized as being lifestyle-related. The incidence of gout and related hyperuricemia is increasing yearly [34]. Gout is caused by an increase in serum uric acid levels, which is the final metabolite of purine catabolism in humans. Inhibiting XO is one of the effective methods to lower the level of serum uric acid [2,3]. It has been shown that naturally occurring phytochemicals such as polyphenols possess XO-inhibitory properties, are effective XO inhibitors that lower the production of uric acid, and are considered potential therapeutic agents to prevent and treat gout. *Smilax china* L. polyphenols exhibit anti-obesity, anti-inflammatory, and anti-bacterial effects [20,21,22,23]. In this study, *Smilax china* L. had a good inhibitory effect on XO, with the ethyl acetate-soluble parts in particular having the best effect, which might be related to polyphenols and other active components. It has been confirmed that the ethyl acetate-soluble parts have the highest contents of polyphenols (Figure 1). 

In our current study, the three compounds identified by HPLC were flavonoids and glycosides. Quercetin-3-O-rhamnoside, known as quercitrin, is a well-known flavonoid compound that exerts anti-inflammatory and antioxidant activities [35]. Engeletin is a flavonoid glucoside substance. Studies have shown that engeletin has anti-inflammatory effects and improves the inflammatory symptoms related to endometritis by inhibiting the activation of NF-κB [36]. Wei et al. showed that engeletin improves diabetes complications and inhibits LPS-induced inflammation [37]. Likewise, isoquercetin (IC_50_ value of 0.185 mmol/L) was reported to demonstrate great α-glucosidase-inhibitory effects, inhibiting α-glucosidase activity in a non-competitive manner [38]. Recently, an in vitro and in silico study reported that isoquercetin from red onion (*Allium cepa* L.) solid waste exhibited potent xanthine oxidase enzyme-inhibitory activity [39].

Thirteen compounds in the EtOAc fraction were detected by HPLC-MS. Liquid phase and mass spectrometry is one of the most widely used methods for composition identification, particularly for identifying the active components of natural products. Researchers have reported that polyphenol has been shown to be more effective in inhibiting XO than allopurinol, with an IC_50_ value of 0.274 μM for XO and one of 4.784 μM for allopurinol [40]. Chlorogenic acid is an effective XO inhibitor; it binds to sites other than the active site through mixed-type inhibition [41]. According to the results reported by Zhou et al. [42], chlorogenic acid ameliorates hyperuricemia in vivo. In this study, the inhibition of the EtOAc fraction on XO was reversible and competitive, indicating that the EtOAc fraction reduced the enzyme activity by competing with the active center of XO, thus reducing the production of uric acid. Nevertheless, in this work, we did not use monomers to examine their efficacy. As a result, we are unable to distinguish the actual specific effect of each single compound. It is still necessary to conduct relevant experiments in vitro and in vivo to determine whether the *Smilax china* L. polyphenols are suitable candidates to treat gout.

## 4. Materials and Methods

### 4.1. Materials

Xanthine oxidase, xanthine, and standard substances (≥98%) including chlorogenic acid, gallic acid, quercetin-3-O-rhamnoside, kaempferol, quercetin, kaempferitrin, engeletin, iso-quercetin, astragalin, and rutin were bought from Beijing Solarbio Technology Co., Ltd. (Beijing, China). Allopurinol was supplied by Shanghai Xinyi Wanxiang Pharmaceutical Co., Ltd. (Shanghai, China). A laboratory mill was purchased from Zhejiang Rhodiola Industry and Trade Co., Ltd (DE-1000gA, Jinhua, China). The qualitative analysis of compounds was completed by HPLC analysis on an Agilent 1260 HPLC system (Agilent Technologies, Santa Clara, CA, USA) equipped with a C18 reverse-phase column.

### 4.2. Preparation of the Extracts

*Smilax china* L. root was brought from a Simcere drugstore in Nanjing, China. The dried root of *Smilax china* L. was ground into a fine powder with a laboratory mill and ultrasonically extracted with 95% ethanol (1:20) for 40 min. After concentration, the crude ethanol extract was obtained with a yield of 9.2%. The ethanol extract was further fractionated according to solvent polarity. The ethanol extract was then resuspended in petroleum ether (PE), chloroform, EtOAc, and n-butanol (n-BuOH) in order of increasing polarity at room temperature with occasional shaking using a separating funnel. After that, these fractions were centrifuged, filtered, and concentrated to obtain PE, chloroform, EtOAc, n-BuOH, and residual ethanol solution fractions. The extraction yields of the PE, chloroform, EtOAc, n-BuOH, and residual ethanol fractions were 0.64 ± 0.04%, 1.18 ± 0.07%, 5.02 ± 0.23%, 2.82 ± 0.15%, and 0.54 ± 0.04% (*w*/*w*, on a dry-weight basis), respectively. Finally, these extracts were concentrated, freeze-dried, and then stored in a desiccator until use. The total phenolic content was measured using the Folin–Ciocalteu method, and the total flavonoid content was determined by a colorimetric method [43].

### 4.3. XO-Inhibitory Activity

The XO inhibition assay was carried out by the methods used by Zhou et al. [44] with minor modifications. In brief, in a 200 μL enzyme reaction system, 60 μL of sodium phosphate buffer (pH = 7.5), 30 μL of XO solution (0.1 U/mL), and various concentrations of ethanol extracts (50 μL) were added to a 96-well plate, and the mixture was incubated for 15 min at 25 °C. Then, the reaction was started by adding 60 μL of xanthine solution (150 μM) to the mixture as a substrate. After incubating, the absorbance was measured at 295 nm. Allopurinol was used as a positive control. The inhibition ratio (%) was calculated as follows:

Inhibition ratio (%) = (1 − A/B) × 100%

where A is the reaction rate of the system containing the inhibitor; B is the reaction rate of the system without the inhibitor. The extent of inhibition was expressed as IC_50_ (half of the maximum effective concentration).

### 4.4. Lineweaver–Burk Plots

In the reaction system between the inhibitor and enzyme, the concentration of fixed substrate was 150 μM, and the reversibility of enzyme inhibition was measured by varying concentrations of XO and different amounts of inhibitor. The curves of the reaction rate (*v*) vs. [XO] were depicted to assess the reversibility.

### 4.5. Inhibition Type of the EtOAc Fraction on XO by Kinetic Analysis

Different concentrations of EtOAc fraction solutions were prepared (0, 80, and 160 μg/mL), the reaction conditions remained constant, and the concentration of XO was fixed at 0.1 U/mL. The relationship between the reaction rate and substrate concentration was determined under different inhibitor concentrations. The inhibition type was analyzed according to the Lineweaver–Burk plot. For competitive inhibition, the Lineweaver–Burk equation can be written as [29]:

(1) $$\frac{1}{v}=\frac{K_{m}}{V_{max}}\left(1+\frac{\left[I\right]}{K_{i}}\right)\frac{\left[1\right]}{\left[S\right]}+\frac{1}{V_{max}}$$

(2) $$K_{m}^{app}=\frac{K_{m}[I]}{K_{i}}+K_{m}$$

where *K_i_* and *K_m_* denote the inhibition constant and Michaelis–Menten constant, respectively, and *v* is the enzyme reaction rate. [*I*] and [*S*] are the concentrations of the inhibitor and substrate, respectively. $K_{m}^{app}$ represents the apparent Michaelis–Menten constant.

### 4.6. HPLC–MS Analysis

The EtOAc extracts were subjected to HPLC apparatus separation on an Agilent 1260 HPLC system (Agilent Technologies, Santa Clara, CA, USA) equipped with a series of SHODEX KS-804 and KS-802 columns (8 mm × 300 mm) and a refractive index detector. The mobile phase was acetonitrile (solvent A) and 0.1% formic acid aqueous solution (solvent B). The linear gradient solvent system was as follows: 0–10 min, 15–25% A; 10–22 min, 25–31% A; 22–30 min, 31–45% A; 30–31 min, 45–100% A; and, finally, isocratic elution with 100% phase A until 35 min. The column temperature was 20 °C, the injection volume was 10 μL, the detection wavelength was 280 nm, and the flow rate was maintained at 0.8 mL/min. The MS analysis was performed using an Agilent Technologies 6538 OHD accurate-mass quadrupole time-of-flight (Q-TOF) mass spectrometer to analyze and identify the EtOAc fraction’s active compounds. The Q-TOF mass spectrometer was equipped with an electrospray ionization source (ESI) in the negative mode. The operating parameters were set as follows: spray voltage of 4000 KV, gas collision at He, nebulizer pressure at 50 psi, collision voltage of 250 V, and drying temperature of 350 °C. The ion scanning range was 100–1000 *m*/*z*. Data from Agilent’s MassHunter 8.0 software were analyzed.

### 4.7. Statistical Analysis

The experimental results were expressed as mean ± standard deviation (SD) and analyzed by one-way ANOVA using the Origin 8.5 software; *p* < 0.05 was considered significant.

## 5. Conclusions

In this study, *Smilax china* L. ethanol extracts exhibited an inhibitory effect on XO, especially the EtOAc fraction. The EtOAc fraction showed excellent XO-inhibitory performance in the competitive mode. Sixteen compounds in the EtOAc fraction were identified by HPLC and HPLC-MS. Thus, the ethanol extract of *Smilax china* L. has great potential for inhibiting XO activity and preventing hyperuricemia. Future studies should focus on the pharmacological testing of these compounds in vivo.

## Figures and Tables

**Table 1:** The inhibitory kinetic parameters of the EtOAc fraction on XO.

EtOAc Fraction (μg/mL)	*K_m_* (μmol/L)	*K_i_* (μg/mL)	*V_max_* (ΔOD/min)
0	26.32		0.052
80	71.43	46.67	0.052
160	90.91	65.20	0.052

**Table 2:** The single crystal in the EtOAc fraction.

RT (min)	Name	CAS RN	Molecular Weight	Formula	Peak Area (%)
15.683	Isoquercetin	482-35-9	464.38	C_21_H_20_O_12_	0.9500
18.457	Quercetin-3-O-rhamnoside	522-12-3	448.38	C_21_H_20_O_11_	6.5736
20.283	Engeletin	572-31-6	434.39	C_21_H_22_O_10_	2.4612

**Table 3:** The main constituents of the EtOAc fraction detected using HPLC-MS.

No.	Rt (min)	MS [M−H]^−^	Calculated Molecular Weight	(ppm)	Molecular Formula	MS/MS Fragments	Proposed Compounds
1	3.3	353.0859	354.0951	5.38	C_16_H_18_O_9_	191.055 (100), 192.053 (5.33)	Chlorogenic acid
2	3.57	329.0861	330.0951	1.71	C_14_H_18_O_9_	167.035 (100), 168.037 (7.25), 123.044 (11.27)	Vanillic acid hexose
3	3.85	289.0691	290.079	9.18	C_15_H_14_O_6_	121.026 (100), 109.033 (57.83), 125.024 (61.56)	(−)-Epicatechin or catechin
4	3.95	739.1612	740.1682	−0.31	C_46_H_28_O_10_	569.108 (16), 449.088 (12), 435.074 (13), 339.050 (35), 289.070 (100), 245.080 (18), 177.019 (54),	Catechin-O-digalloyl-C-rhamnoside
5	4.2	329.0856	330.0951	6.68	C_14_H_18_O_9_	123.046 (16), 167.0333 (100), 191.031 (14), 209.043 (10)	Vanillic acid hexose isomer
6	4.57	739.1609	740.1682	0.07	C_46_H_28_O_10_	289.070 (100), 245.083 (27.42), 217.010 (8.11), 177.018 (94.57), 325.027 (7.07), 339.050 (48.53), 459.068 (33.07)	Catechin-O-digalloyl-C-rhamnoside isomers
7	5.36	449.1062	450.1162	4.52	C_21_H_22_O_11_	151.003 (100), 285.039 (48.96)	Eriodictyol-C-hexose
8	5.97	449.1055	450.1103	−5.42	C_28_H_18_O_6_	151.003 (100), 179.010 (18.04), 199.038 (26.38), 259.058 (14.75), 285.038 (57.16)	Eriodictyol-C-hexose isomers
9	6.2	451.0994	452.1049	−4.0	C_24_H_20_O_9_	341.064 (100), 231.031 (13.07), 217.013 (62.22), 189.017 (36.88), 177.017 (35.16)	Cinchonain I isomer
10	6.99	243.0635	244.0695	−5.08	C_9_H_12_N_2_O_6_	175.073 (100), 99.072 (61.89)	Uridine
11	8.37	451.0994	452.1049	0.18	C_24_H_20_O_9_	341.063 (100), 231.029 (35), 217.011 (79), 189.017 (62), 177.018 (36), 151.040 (22), 109.026 (42)	Cinchonain I
12	9.46	613.1278	614.1352	0.21	C_37_H_14_N_10_O	341.061 (100), 503.092 (52), 393.060 (43), 379.031 (22)	Cinchonain I hexose
13	10.18	227.068	228.076 228.0746	2.99 −2.87	C_9_H_12_N_2_O_5_	183.076 (7.05), 159.083 (20.33), 144.051 (12.54), 143.048 (100), 119.047 (8)	(2)-2-(Aspartylamino)-4-pentynoic acid

Note: RT, retention time; MS: precursor ion obtained from mass spectrum; MS/MS: fragment ions of precursor ion obtained from tandem mass spectrum.

## Data Availability

Not applicable.
